# Vygotsky’s Legacy: Understanding and Beyond

**DOI:** 10.1007/s12124-021-09652-6

**Published:** 2021-10-02

**Authors:** René van der Veer

**Affiliations:** Leiden University, Leiden, The Netherlands

**Keywords:** Understanding Vygotsky, Cultural-Historical Theory, History of psychology, Pedology

## Abstract

The article sketches the history of the study of Vygotsky’s legacy in the Soviet Union and the West and then switches to a brief discussion of the origin of the book *Understanding Vygotsky* published 30 years ago. Several features and shortcomings of the book are discussed and it is shown that recent publications partly fill the gaps in our knowledge. This is illustrated by a succinct discussion of the contributions to the special issue which show that Vygotsky’s legacy continues to inspire the modern researcher.

Understanding Lev Vygotsky’s life and work has never been easy. Born in the Tsarist Empire under Nicholas II, he was destined to lead a quiet and comfortable life as a lawyer or journalist when the October Revolution destroyed the society as it was. The Vygodsky family lost its properties and Vygotsky spent the rest of his life in relative poverty like so many of his Soviet compatriots. However, each revolution creates opportunities for some part of the population and there is every reason to believe that Vygotsky came to share the novel ideal of educating the vast masses of illiterate people. He soon became engaged in the new Soviet system of evening courses, correspondence courses, and schools for people with little or no systematic training and knowledge.

The challenges for the educational system during and after the revolution and civil war were enormous: millions of homeless children roamed the streets, teachers were underpaid and/or opposed the new ideas, school facilities were poor, there was a shortage of paper, textbooks, and so on. Creating a new, Soviet, child required new organizations, skills, and knowledge, and the Soviet authorities under the guidance of Lenin’s wife, Krupskaya, soon adopted the discipline of child study or pedology as a major instrument in the reform of the educational system (Van der Veer, 2020). It was within the field of pedology that Vygotsky made his career and it was the ban on pedology in 1936 that temporarily thwarted the spread of his ideas. However, former colleagues and students carefully preserved his books, articles, and lecture notes, which is illustrated in a recent Russian novel that mentions an older woman, who was fired during the attack on pedology “but kept all Vygotsky’s publications as a treasure and only gave them to read to selected people” (Ulitskaya, 2013, p. 84; cf. Grigorenko in this issue for the similar story about Serapion Korotaev).

After the death of Ioseb Besarionis dze Jughashvili, better known under his nickname Stalin, in 1953, things gradually became somewhat better. Three years later, a first collection of Vygotsky’s writings was published in the Soviet Union (Vygotsky, 1956), followed by another publication in 1960 (Vygotsky, 1960). However, publishing Vygotsky’s writings was still considered a politically very delicate affair in the Soviet Union of that time and it would take another twenty years before the plans for the publication of Vygotsky’s collected works ((Vygotsky, 1982a, b, 1983a, b, 1984a, b) finally materialized. Recently, it became known that for some reason Aleksey Leont’ev, Vygotsky’s erstwhile colleague, deliberately and endlessly delayed writing the general introduction to the first volume—and, hence, frustrated the publication of all six volumes—until one of the other editors, Vasiliy Davydov, finally asked Leonid Radzikhovskiy, by that time a PhD student, to write the introduction under Leont’ev’s name (Radzikhovsky, 2020). Although the collected works proved to be marred by all sorts of mistakes, omissions, intrusions, and political censorship, they nevertheless performed a major role in introducing a large body of Vygotsky’s writings to the newer generations of Russian psychologists.

Meanwhile in the West very few people had ever heard of the Soviet pedologist, let alone read one of his relatively scarce foreign publications (Vygotsky & Luria, 1930; Vygotsky, 1925; 1929a, b, c; 1930; 1934a, 1935a, b; 1936; 1939), when an abridged version of his last book (Vygotsky, 1934b) was published in English under the title of *Thought and Language* (Vygotsky, 1962). This book triggered the interest in Vygotsky’s ideas and a curious compilation of several of Vygotsky’s writings, edited by Cole, John-Steiner, Scribner, and Souberman, and published under the title of *Mind in society*, became a major success in the English reading world (Vygotsky, 1978). The interest in Vygotsky’s writings now grew rapidly and interesting interpretations of several aspects of his work appeared (e.g., Kozulin, 1990; Wertsch, 1985) as well as other interpretations and translations of his works in many countries all over the world. However, a more or less comprehensive study of Vygotsky’s life and work that situated his ideas in the context of his time and showed the connections with other thinkers did not yet exist. This was the situation in the mid 1980s when Jaan Valsiner and the present author decided that it would be interesting to write just such a book.

Writing *Understanding Vygotsky* (Van der Veer & Valsiner, 1991) was not an easy task. The internet did not yet exist, books and articles had not yet been digitalized and most Western libraries had just a handful of the needed literature. The repeated visits to the Soviet Union were also somewhat frustrating. The biggest library of the country, the V.I. Lenin State Library of the USSR or *Leninka* in Moscow, limited xeroxes of articles or books to 10 pages per day and ordering xeroxes required at least 2–3 h of queuing. Books or journals could not be taken from the shelves for reading and had to be ordered as well, which involved an equally time-consuming procedure. In sum, one could easily spend a day in the library and achieve less than we can now in one hour. The Russian people with whom we talked (e.g., Vasiliy Davydov, Semyon Dobkin, Pyotr Gal’perin, Tamara Lifanova, Andrey Puzyrey, Leonid Radzikhovskiy, Gita Vygodskaya, Vladimir Zinchenko) were all very friendly and helpful, but the Soviet Union still existed and none of them was in a position to reveal embarrassing secrets to a foreigner. Moreover, one could sense that decades of brutal suppression and the more or less explicit anti-Semitism left their traces in the mind of people. Hence, it is not surprising that Leonid (Lyosha) Radzikhovskiy never revealed to me that he was the author of Leont’ev’s general introductory article nor did it ever cross my naïve mind that this was a possibility that I should ask him about. That Soviet psychology was divided into various rivalling schools did not help either: in the mind of Vygotsky followers, any criticism of his ideas could be exploited by a rival school, for instance the Rubinstein group led by Andrey Brushlinskiy. Against this background, it came as no surprise when Vasiliy Davydov suggested to us not to mention *Studies on the history of behavior: Ape, primitive, and child* (Vygotsky & Luria, 1930, 1993), which he considered to be an outdated book that might do harm to Vygotsky’s reputation.

Nevertheless, in the end the repeated visits to Moscow and the sustained searches in many Western libraries paid off and after some years a sizable list of historical documents and writings enabled us to begin the reconstruction of Vygotsky’s life and work. The idea of the book was to show that in creating his novel theory about the development of the human mind Vygotsky heavily relied on the work of his predecessors and contemporaries. As I wrote recently (Van der Veer, 2021a), Vygotsky was not ‘a visitor from the future’ (Jerome Bruner), nor ‘a researcher whose ideas were ‘ahead of our time’ (Norris Minnick) but a very bright scholar who operated within the constraints of his cultural, social, and political environment. Above all, Vygotsky achieved a novel synthesis of existing ideas adapted to the demands of the new communist society. In *Understanding Vygotsky* we tried to give various examples of the interconnectedness of Vygotsky’s ideas with those of his contemporaries.

The chapter on Gestalt psychology, for example, showed how Vygotsky used the non-reductionist ideas of Köhler, Lewin, Koffka, and Goldstein and at the same time resisted their non-dialectical approach to human development. Recently, about twenty-five years later, Anton Yasnitsky (e.g., Yasnitsky and Van der Veer, 2016) again drew attention to the link between Vygotsky’s theorizing and the ideas advanced by Gestalt psychologists, although his use of the term ‘cultural-historical Gestalt psychology’ to designate Vygotsky’s ideas is surely exaggerated.

Another chapter that introduced a new perspective to the then existing view of Vygotsky highlighted his role in the discipline of pedology, which, as said above, had been adopted by the Soviet authorities as the leading science within the educational system. With contemporaries such as Mikhail Basov, Pavel Blonskiy, Stepan Molozhavyy, and Nikolay Shchelovanov, Vygotsky carried out pedological investigations and tried to define the subject matter of the new discipline and its place amidst neighboring disciplines such as developmental psychology, educational psychology (pedagogics), and pediatrics. As we argued at the time, pedology allowed Vygotsky to combine the study of the development of novel complex functions with that of the educational needs of normal and retarded children. The focus on Vygotsky the pedologist was novel at the time and the Soviet history of pedology was still little known in Russia and elsewhere. It is only recently that Byford has deepened the study of the history of this discipline in several excellent publications (e.g., Byford, 2014, 2016, 2021; cf. Van der Veer, 2020).

Yet another chapter focused on Vygotsky and Luria’s involvement in psychoanalysis. Again, this was a story that was largely unknown to the followers of Vygotsky and one which Soviet historians preferred to ignore given that psychoanalysis had become one of the many forbidden disciplines around 1930. Luria’s active participation in the international psychoanalytic movement and Vygotsky’s more distant involvement were sketched against the tragicomic background of the development and demise of Freudo-Marxism. Several years later, Alexander Etkind (1997) would considerably enrich this story in his fascinating account of the history of psychoanalysis in Russia.

All in all, *Understanding Vygotsky* asked the reader to consider the links between Vygotsky’s ideas and the web of other ideas available to him in order to understand his intellectual creativity in its historical context. But, of course, the book had many lacunae. We did not know much about Vygotsky’s personality and his personal life, for example. Here the book by his daughter Gita Vygodskaya and Tamara Lifanova, published five years later, proved of great value (Vygodskaya & Lifanova, 1996). Neither did we know the finer details of the cross-cultural psychological expedition to Uzbekistan headed by Luria, how Vygotsky practiced clinical work with children, how much he had written about Jewish issues, and many, many other things.

It is here that almost twenty years later the publication and analysis of Vygotsky’s notebooks by Ekaterina Zavershneva proved of fundamental importance. In a series of publications (e.g., Zavershneva, 2010a, b, c), she shed light on many of Vygotsky’s preoccupations and his habit to return time and again to the fundamental problems of psychology (e.g., the relationship between thinking and speech). These writings finally culminated in the publication of a major amount of Vygotsky’s notes in *Vygotsky’s Notebooks: A selection* (Zavershneva & Veer, 2017, 2018). The potential of this volume remains yet to be explored (cf. Kölbl & Métraux, 2021; Maidansky, 2020), but it seems clear that the published notes substantially enrich our picture of Vygotsky the scientist and the man. The early notes are particularly interesting, because they show the young Vygotsky (i.e., around 1916–1917, when the nation was already falling apart) to be very much involved with the issue of Jewish identity (see Zavershneva and Veer, 2018; chapters 1 to 4). A later notebook gives a unique insight into Vygotsky’s mood swings and personality. Written in 1925, during his only trip abroad, the notebook shows a rather neurotic young man who is obsessively taking notes, describing his feelings of loneliness, depression, agitation, anxiety, etc., in a foreign country, and is longing for his wife and newborn child (see chapter 8). A much later notebook, written around 1933–1934, contains nine case histories of children seen by Vygotsky and his colleagues. Vygotsky’s accounts are quite moving, because they clearly show how these ‘difficult’ children were unable to cope with virtually impossible life circumstances and ended up as social misfits and mental patients in the hands of psychiatrists and clinical psychologists (see chapter 27). In sum, there is still much in the 29 chapters of this collection that remains to be analyzed and Zavershneva deserves full credit for the exploration of Vygotsky’s family archive. And yet, of course, even after the necessary analysis of the findings in *Vygotsky’s Notebooks* many issues of Vygotsky’s legacy will still be awaiting discussion and clarification, which is beautifully shown by the contributions to this special issue to which I now turn.

Luciano Mecacci author points out that for Vygotsky man was a ‘political animal’, i.e., a being living in a concrete and dynamic social-cultural network, which immensely varies both within and between human cultures. If such social-cultural networks or practices offered insufficient opportunities to the person, psychologists should attempt to apply their knowledge to try to change these practices (‘to modify the social reality and consequently the psychological life of people’). In Vygotsky’s view, the success or failure of such attempts proves or refutes the validity of the scientific knowledge and thus forms its ultimate truth criterion. Many years later, this view appealed to various progressivist groups who advanced ‘critical’ psychologies (cf. Van IJzendoorn & Van der Veer, 1984). An example can be found in the field that studies children who are challenged in their development; these shouldn’t be judged by their difference from mainstream norms, which are essentially based on the behavior and mindset of a dominant group. The *general* child doesn’t exist and extrapolating norms that hold in one (sub)culture to others leads to misguided judgments and injustice. As Mecacci points out, comparing children and adults from different groups (e.g., rural versus urban subjects) was already a hot topic among pedologists in the Soviet Union of the 1920s and 1930s. In fact, after fierce debates, it was decided that mental tests that used norms established in certain dominant groups should no longer be applied in other groups given the inherent difficulties (Van der Veer, 2021b).

That these difficulties are still among us is beautifully illustrated by Elena Grigorenko. In her research, inspired by Vygotsky’s pedological writings, she explicitly attempts to contextualize her concepts and instruments and seeks to uncover the subjects’ potential for further development. One means to create subjects’ zone of proximal development is dynamic testing that is specifically alert to cross-cultural differences, as Grigorenko demonstrates. In my view, such research also exemplifies Vygotsky’s dictum that we should not identify children with special needs with their handicap or challenge. Both normal and ‘abnormal’ children should not be characterized by their present state but by their potential for further development.

Pablo del Río and Amelia Álvarez point out that according to Vygotsky’s theory cultural changes bring about changes in people’s minds and that external cultural mediation allows human beings to transcend the here and now. Such changes necessarily correspond with modifications of the brain’s organization for which modern neurological research has found ample evidence. The authors also point out that Vygotsky’s more personal quest seems to have been that for freedom in a psychology of heights or acmeist psychology. Acmeist psychology they connect with Vygotsky’s older ideal to create a new human being, which anticipated modern fantasies about improving the human brain and body with various technologies. In the authors’ view, cultural-historical psychology should participate in the debate about the feasibility and goal of redesigning the human subject.

Acmeist psychology, a term he connects with Vygotsky’s affinity with poetry, is also the topic of Carlos Kölbl’s contribution. The author attempts to outline acmeist psychology by reflecting on three methodological principles that Vygotsky discussed. The objective-analytical method rests on the study of a single phenomenon that is claimed to be representative for a whole class of phenomena. The method of double stimulation involved the introduction of signs into the experimental situation that subjects could use to solve a task. In *Understanding Vygotsky* we argued that this method was based on Wolfgang Köhler’s famous chimpanzee experiments but Kölbl interestingly suggests that Vygotsky also may have thought of Velimir Khlebnikov’s radical poetry. Finally, the semic method involves the study of the fluctuating values of meaning and sense in development. The author argues that these methodological principles were all inspired by and applicable to phenomena of art but may also serve as inspiration for the creation of a future scientific psychology.

Nikolay Veresov argues that cultural-historical theory is still insufficiently understood and suggests that newer publications may serve to understand its dialectical nature. Veresov argues against the common misunderstanding that lower functions disappear or get transformed by higher functions. For example, adult human perception does not differ from neonatal or animal perception in that the primary visual system (located in the occipital lobe) is transformed or stops functioning. Rather, what happens is that language, knowledge, and motivation (located in the parietal, temporal, and frontal lobe) together with the visual system make another type of recognition and classification of the outside world possible. As was written in *Understanding Vygotsky*, adult human beings live in a world of meanings or a *semantic universe*. What Veresov seeks to do in his paper is to detail the exact dialectical relationships between lower and higher functions that create human adult functioning.

In *Understanding Vygotsky* we described how Vygotsky worked on a culturally and historically informed theory of emotional development. There is little doubt that adult emotions (e.g., the pleasure felt when composing a chess study or finding a mathematical proof) differ from those of a two-year-old but Vygotsky’s turn to Spinoza and the idea that emotions should be subjected to the control of the intellect did not go uncontested. In his contribution, Peter Smagorinsky contrasts Vygotsky’s view with Jonathan Haidt’s theory that much of human thinking is based on gut feelings rather than logical reason. The author argues that Vygotsky’s theory ultimately suffered from the exportation of an Enlightenment rationality based on Eurocentric ethnocentrism (cf. the papers by Mecacci and Grigorenko). In addition, Vygotsky overestimated the role of language acquisition in the socialization of the child which blinded him to nonverbal forms of communication. Smagorinsky is inclined to prefer Haidt’s view that logical argumentation only serves to justify or rationalize initial intuitions but realizes that arguing such a view would be paradoxical.

Tania Zittoun traces Vygotsky’s use of the cloud-rain metaphor and its use in thinking about different levels of psychological functioning in the notes he kept taking throughout his life. The full metaphor is first used in December 1932 when Vygotsky is exploring the relationship between motivation, thinking, and speech by referring to the image of wind, cloud, and rain. The image allows Vygotsky to think about the transitions between various levels of mental functioning such as speech, inner speech, and thought. For example, whereas ideas or mental images seem to be experienced as a whole (i.e., a cloud), formulating or articulating them in words necessarily involves fragmentation (i.e., drops of water). Zittoun’s chronological analysis of Vygotsky’s use of the metaphor is particularly interesting, because he seems to have been absolutely unable to grasp or feel things without first putting them into words (cf. Smagorinsky’s paper). For Vygotsky, the use of specific words and metaphors definitely served to shape his ideas.

Taken together, the papers in this special issue demonstrate that modern researchers still find inspiration in the work of the Russian pedologist. The idea of *Understanding Vygotsky* never was to glorify his work, which is necessarily constrained by the social and scientific context of his time, but to allow us to understand and extend his ideas and to see whether some variant of them can help us to develop our science further. That this is still possible—as the contributions in this issue amply demonstrate—seems to prove, despite repeated claims to the contrary by Anton Yasnitsky, that the rumors of Vygotsky’s scientific death are greatly exaggerated.
